# Data-Driven Machine-Learning Methods for Diabetes Risk Prediction

**DOI:** 10.3390/s22145304

**Published:** 2022-07-15

**Authors:** Elias Dritsas, Maria Trigka

**Affiliations:** Department of Computer Engineering and Informatics, University of Patras, 26504 Patras, Greece; dritsase@ceid.upatras.gr

**Keywords:** diabetes, prediction, Machine Learning, data analysis

## Abstract

Diabetes mellitus is a chronic condition characterized by a disturbance in the metabolism of carbohydrates, fats and proteins. The most characteristic disorder in all forms of diabetes is hyperglycemia, i.e., elevated blood sugar levels. The modern way of life has significantly increased the incidence of diabetes. Therefore, early diagnosis of the disease is a necessity. Machine Learning (ML) has gained great popularity among healthcare providers and physicians due to its high potential in developing efficient tools for risk prediction, prognosis, treatment and the management of various conditions. In this study, a supervised learning methodology is described that aims to create risk prediction tools with high efficiency for type 2 diabetes occurrence. A features analysis is conducted to evaluate their importance and explore their association with diabetes. These features are the most common symptoms that often develop slowly with diabetes, and they are utilized to train and test several ML models. Various ML models are evaluated in terms of the Precision, Recall, F-Measure, Accuracy and AUC metrics and compared under 10-fold cross-validation and data splitting. Both validation methods highlighted Random Forest and K-NN as the best performing models in comparison to the other models.

## 1. Introduction

Diabetes mellitus is a common metabolic disease characterized by high blood glucose levels. In diabetes, the body inefficiently produces little or no insulin. Increased blood sugar (hyperglycemia) and impaired glucose metabolism occur either as a result of decreased insulin secretion or due to decreased sensitivity of the body cells to the action of this hormone (insulin) [1]. Depending on the insulin disorder, diabetes is classified into the following types:

- **Type I diabetes or juvenile diabetes**: In this type, insulin-producing pancreatic cells are destroyed by an autoimmune mechanism (that is, by antibodies produced by the body itself). It mainly affects young people, insulin is completely absent, and the patient requires insulin therapy from the beginning [2].
- **Type II diabetes**: It is characterized by increased resistance of the body to insulin with the result that what is produced is not sufficient to meet the metabolic needs of the body. Type 2 diabetes is the most common cause of diabetes in adults. An important predisposing factor for the development of type 2 diabetes is obesity. Other predisposing factors are age and family history. If necessary, anti-diabetic drugs are used. In case the treatment fails, it is recommended to administer insulin to control these patients as well [3].
- **Gestational diabetes**: It is a type of diabetes that first appears during pregnancy (excluding women with pre-pregnancy diabetes). This type is similar to type 2 diabetes. Obese women are more likely to develop gestational diabetes. Gestational diabetes is reversible and resolves after childbirth but can cause perinatal complications and maternal and neonatal health problems [4].

Diabetes often has no symptoms. If they do occur, the symptoms may include thirst, frequent urination, overeating and hunger, fatigue, blurred vision, nausea, vomiting and weight loss (despite overeating) [5]. Some people are more likely to develop diabetes. Various factors may be taken into consideration to evaluate the associated risk for its occurrence. In particular, people who are more prone to develop diabetes are usually over 45 years and physically inactive in their daily life.

From a gender and waist perspective, men with a waist circumference greater than 102 cm or women with a waist circumference greater than 88 cm have a higher risk for developing diabetes. Furthermore, a body mass index greater than 30 is an indicator of obese people. Finally, diabetes relates to the coexistence of other comorbidities, such as elevated cholesterol levels, history of diabetes in the immediate family environment, hypertension or cardiovascular disease, peripheral vascular disease, women with polycystic ovaries, gestational diabetes (especially women who are pregnant with overweight children) and drugs that cause diabetes (e.g., cortisone) [6,7].

Chronic complications of diabetes can be reduced through regular blood sugar control. The target organs affected by diabetes are the eyes, the kidneys, the nervous system and the vessels of the heart, brain and peripheral arteries [8,9].

Early diagnosis of the disease is crucial to avoid unpleasant developments regarding the patient’s health. Lifestyle changes with proper diet and exercise, as well as medication under the supervision of appropriate physicians, are the most important elements for an effective therapeutic approach. The science of medicine has made great steps in reducing disease mortality and improving patients’ quality of life [10,11].

Proper treatment of patients with diabetes is imperative currently as we deal with the critical pandemic COVID-19. It should be noted here that patients with diabetes are more likely to have complications from COVID-19 and have increased mortality [12].

Recent advances in the fields of Artificial Intelligence (AI) and Machine Learning (ML) may provide clinicians and physicians with efficient tools for the early diagnosis of various diseases, such as Cholesterol [13], Hypertension [14], COPD [15], Continuous Glucose Monitoring [16], Short-Term Glucose prediction [17], COVID-19 [18], CVDs [19], Stroke [20], CKD [21], ALF [22], Sleep Disorders [23], Hepatitis [24] and Cancer [25]. The prediction of type 2 diabetes is the point of interest in this research work. For this specific disease, numerous research studies have been conducted with the aid of machine-learning models.

For the purpose of the specific research, we present a type 2 diabetes risk assessment framework consisting of a plethora of classification models and assuming as risk factors the gender, age (demographic data) and the most common symptoms that relate to the diabetes development. The contributions of this manuscript are two-fold. First, after class balancing, features analysis is conducted, which includes (i) feature ranking to identify their order of importance in the diabetes class and (ii) capturing their prevalence in the diabetes class.

The second proposition of this paper is a comparative evaluation of several models in order to identify the ones with the highest performance metrics, which means that they are the most appropriate to correctly identify those at high risk. The most common performance metrics are utilized to evaluate the classifiers’ performance, such as the Precision, Recall, F-Measure, Accuracy and AUC. Performance analysis is conducted after the application of class balancing, assuming 10-fold cross-validation and data splitting, which demonstrated that Random Forest and K-NN are the most efficient models.

They achieved an accuracy of 98.59% after SMOTE with 10-fold cross-validation and 99.22% after SMOTE with a percentage split (80:20) in comparison to the other models. Furthermore, the proposed models were compared with published research works that used the same dataset with the same features we relied on. From the results of the experiments, our models outperformed in all cases.

The rest of the paper is organized as follows. Section 2 describes the relevant works with the subject under consideration. In addition, in Section 3, a dataset description and analysis of the methodology followed are made. In addition, in Section 4, we discuss the acquired research results. Finally, our conclusions and future directions are outlined in Section 5.

## 2. Related Work

Currently, researchers have paid great attention to the development of AI-based tools and methods suitable for chronic conditions monitoring and control. Specifically, ML models have been widely utilized to quantify the risk of a disease occurrence assuming various features or risk factors. In the context of this section, our purpose is to present relevant works concerning diabetes.

First, the authors in [26] proposed a framework for diabetes prediction consisting of different machine learning classifiers, such as K-Nearest Neighbor, Decision Trees, Random Forest, AdaBoost, Naive Bayes and XGBoost and Multilayer Perceptron neural networks. Their proposed ensembling classifier is the best performing classifier with the sensitivity, specificity, false omission rate, diagnostic odds ratio and AUC of 0.789, 0.934, 0.092, 66.234 and 0.950, respectively.

Moreover, in [27], the authors utilized machine-learning techniques in the Pima Indian diabetes dataset to develop trends and detect patterns with risk factors using the R data manipulation tool. They applied supervised machine learning algorithms, such as linear kernel Support Vector Machine (SVM-linear), radial basis function, K-Nearest Neighbor, Artificial Neural Network and Multifactor Mimensionality Reduction, in order to classify the patients into diabetic and non-diabetic. The SVM-linear model provides the best accuracy of 0.89 and precision of 0.88. On the other hand, the K-NN model provided the best recall and F1 score of 0.90 and 0.88, respectively.

In addition, the authors in [28] compared machine-learning-based models, such as Glmnet, Random Forest, XGBoost and LightGBM, to commonly used regression models for the prediction of undiagnosed type 2 diabetes. With six months of data available, a simple regression model performed with the lowest average Root Mean Square Error of 0.838, followed by Random Forest (0.842), LightGBM (0.846), Glmnet (0.859) and XGBoost (0.881). When more data were added, Glmnet improved with the highest rate (+3.4%).

Logistic Regression, K-Nearest Neighbor, Support Vector Machine, Naïve Bayes, Decision Tree and Random forest were applied in [29]. The 10-fold cross-validation was also applied to test the effectiveness of different models. The experimental results showed that the accuracy of Random Forest was 94.10% and outperforms the other models.

Additionally, in [30] Logistic Regression is used to identify the risk factors for diabetes based on *p*-value and odds ratio (OR). The Naïve Bayes, Decision Tree, Adaboost and Random Forest were applied to predict the diabetic patients. Furthermore, three types of partition protocols (K2, K5 and K10) were considered and repeated in 20 trials. The overall ACC of the ML-based system is 90.62%. The combination of Logistic Regression-based feature selection and Random Forest-based classifier gives 94.25% ACC and 0.95 AUC for the K10 protocol.

Furthermore, in [31], dataset creation, features selection and classification using different supervised machine-learning models, such as Naïve Bayes, Decision Trees, Random Forests and Logistic Regression, were considered. The ensemble Weighted-Voting-Logistic Regression-Random Forest ML model was proposed to improve the prediction of diabetes, scoring an Area Under the ROC Curve (AUC) of 0.884.

Finally, published works [32,33,34,35] based on [36] dataset. Specifically, in [32] the authors based on Naive Bayes, Logistic Regression and Random Forest algorithms and, after applying 10-fold cross-validation and percentage split (80:20) evaluation techniques, Random forest has been found to have the best accuracy in order to predict diabetes in both cases. In [33], the authors applied Bayes Network, Naïve Bayes, J48, Random Tree, Random Forest, K-Nearest Neighbor and Support Vector Machine, and, after applying 10-fold cross-validation, the K-Nearest Neighbor performed the highest accuracy with 98.07%.

In [34], Naive Byes, Random Forest, Support Vector Machine and Multilayer Perceptron were applied. The results showed that the Random Forest provides the highest values of 0.975 for precision, recall and F-measure, respectively. Multiplayer perceptron also works well with 0.96 precision value, 0.963 recall value and 0.964 F-measure value, respectively. Last, in [35], the authors based on Artificial Neural Network and Random Forest, and after applying 10-fold cross-validation, the Random Forest outperformed with an accuracy of 97.88%. To sum up, in Table 1 we summarize the aforementioned related works.

## 3. Materials and Methods

In this section, our analysis will focus on the dataset description, the adopted methodology (i.e., data preprocessing, feature ranking and analysis in terms of the target classes), the risk prediction models and the evaluation metrics.

### 3.1. Dataset Description

Our experimental results were based on [36] dataset. No specific processing was performed on this dataset as there were no missing and extreme values. The number of participants is 520 and all the attributes (16 as input to machine-learning models and 1 for the target class) are analyzed as follows:

- **Age** (years) [37]: This feature captures the participant’s age.
- **Gender** [38]: This feature refers participant’s gender. The number of men is 328 (63.1%) while the number of women is 192 (36.9%).
- **Polyuria** [39]: This feature captures whether the participant experienced excessive urination or not. The percentage of participants who had excessive urination is 49.6%.
- **Polydipsia** [39]: This feature captures whether the participant experienced excessive thirst/excess drinking or not. The percentage of participants who experienced excessive thirst/excessive alcohol consumption is 44.8%.
- **Sudden weight loss** [40]: This feature captures whether the participant had an episode of sudden weight loss or not. The percentage of participants who had an episode of sudden weight loss is 41.7%.
- **Weakness** [41]: This feature captures whether the participant had an episode of feeling weak. The percentage of participants who had an episode of feeling weak is 58.6%.
- **Polyphagia** [42]: This feature captures whether the participant had an episode of excessive/extreme hunger or not. The percentage of participants who had an episode of excessive/extreme hunger is 45.6%.
- **Genital thrush** [43]: This feature captures whether the participant had a yeast infection or not. The percentage of participants who had a yeast infection is 22.3%.
- **Visual blurring** [44]: This feature captures whether the participant had an episode of blurred vision or not. The percentage of participants who had an episode of blurred vision is 44.8%.
- **Itching** [45]: This feature captures whether the participant had an episode of itch. The percentage of participants who had an episode of itching is 48.7%.
- **Irritability** [46]: This feature captures whether the participant had an episode of irritability. The percentage of participants who had an episode of irritability is 24.2%.
- **Delayed healing** [47]: This feature captures whether the participant had a noticed delayed healing when wounded or not. The percentage of participants who had noticed delayed healing when wounded is 46%.
- **Partial paresis** [48]: This feature captures whether the participant had an episode of weakening of a muscle/group of muscles or not. The percentage of participants who had an episode of weakening of a muscle/group of muscles is 43.1%.
- **Muscle stiffness** [49]: This feature captures whether the participant had an episode of muscle stiffness. The percentage of participants who had an episode of muscle stiffness is 37.5%.
- **Alopecia** [50]: This feature captures whether the participant experienced hair loss or not. The percentage of participants who experienced hair loss is 34.4%.
- **Obesity** [51]: This feature captures whether the participant can be considered obese or not. The percentage of participants who are considered obese is 16.9%.
- **Diabetes**: This feature refers to whether the participant has been diagnosed with diabetes type 2 or not. The percentage of participants who suffer from diabetes type 2 is 61.5%.

All the attributes are nominal except for age, which is numerical.

### 3.2. Diabetes Risk Prediction

Machine-learning models, more than ever, constitute an important tool for physicians, clinicians and health carers as they allow them to automate the risk assessment of a disease occurrence based on several risk factors. Here, the long-term risk of diabetes development is formulated as a classification task with two target classes c = “Diabetes” (diabetes occurrence) or c = “Non-Diabetes” (non-occurrence of diabetes). The trained ML models will be able to predict the class of an unlabeled instance either as Diabetes or Non-Diabetes based on the input features’ values, and thus the risk of developing diabetes. The main steps of the adopted methodology include data preprocessing, feature ranking, classification models training and performance evaluation.

#### 3.2.1. Data Preprocessing

For the development of efficient models suitable for the accurate identification of Diabetes and Non-Diabetes instances, the non-uniform class distribution was tackled by employing SMOTE [52]. SMOTE method, based on a 5-NN classifier, was used to create synthetic data based on 60% of the minority class, i.e., Non-Diabetes, such that the instances in the two classes are equally distributed (i.e., 50%–50%). This technique is followed to avoid overfitting as it creates new synthetic similar data from the minority class, which are not duplicate or replicate of existing minority class data. Then, the synthetic instances are added to the original dataset.

#### 3.2.2. Features Importance

Four ranking methods were applied to evaluate the contribution of a feature in the target class. Their results are summarized in Table 2.

As for the first method, namely Pearson correlation coefficient [53], it is used to infer the strength and direction of the association between the features and the target class and varies between −1 and 1. More specifically, we observe that a strong correlation of 0.7046 is captured between diabetes and the symptom of polyuria. Furthermore, a moderate relationship of rank 0.6969, 0.5017 and 0.4922 is noted between polydipsia, sudden weight loss and gender with diabetes. The same holds for partial paresis feature and diabetes with a rank of 0.4757. A weaker association is shown to have diabetes with the features of polyphagia, irritability, alopecia, visual blurring and weakness, while the absence of correlation occurs with the rest features where the rank is lower than 0.2.

Gain Ratio (GR) method [54] was also employed, which is calculated as $GR\left(x\right)=\frac{H(c)-H(c|x)}{H(x)}$, where $H\left(x\right)=-p_{x}log_{2}\left(p_{x}\right)$ (with $p_{x}$ denoting the probability of selecting feature *x*), $H\left(c\right)=-p_{c}log_{2}\left(p_{c}\right)$ (with $p_{c}$ be the probability of selecting an instance in class *c*) and H(c|x) are the entropy of an instance with feature *x*, the entropy of class *c* and the conditional entropy of feature *x* given class *c*, respectively. Gain ratio is used to determine the relevance of a feature and chooses the ones that achieve the maximal gain ratio considering the probability of each feature value. Gain ratio, also known as Uncertainty Coefficient, normalizes the information gain (H(c)−H(c|x)) of a feature against how much entropy that feature has.

Furthermore, the Naive Bayes and Random Forest classifiers were selected to measure the importance of the features. Random Forest creates a forest of trees, and per tree measures a candidate feature’s ability to optimally split the instances into two classes using the Gini impurity [55]. Naive Bayes calculates the probability of each feature p(x|c) in order to evaluate their performance at predicting the output variable.

We observe that Naive Bayes and Pearson correlation coefficients assigned the same order of importance in all features except for the age and genital thrush, which are presented in reverse order. Although these methods compute the importance differently, they result in the same ordering outcomes. The same order may relate to the fact that (i) Naive Bayes supposes features independence, as their correlation may harm its performance and (ii) the correlation coefficient measures the strength of each feature’s relationship with the target class [56].

The features of polydipsia and polyuria are unanimously categorized first while features of muscle stiffness, obesity, delayed healing and itching are last in the order by all methods. In the rest features, we observe similarities in the ranking order between different methods. In conclusion, since all features are among the most common symptoms for diabetes screening by physicians (including the blood test for verification), the models’ training and validation will be based on all of them.

#### 3.2.3. Features Exploration

In this section, we aim to present the diabetes prevalence in terms of the involved features. The selected features are among the signs of diabetic patients. The mean age of participants is 47.7 years, and its standard deviation is 12.2.

**Figure 1:**
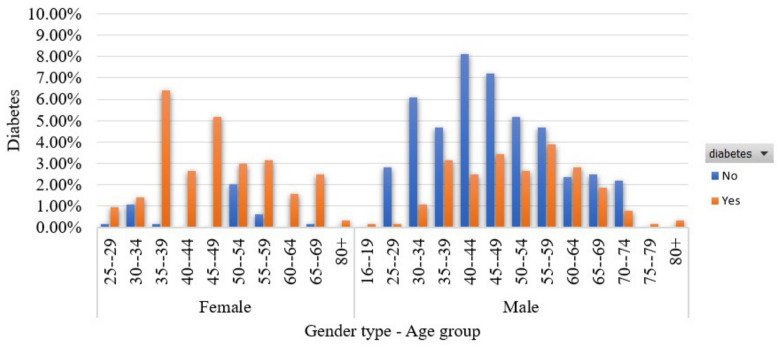
Participants’ distribution in terms of the age group and gender.

In Figure 1, we show the participants’ distribution from both the age group and the gender perspective. We see that most of the involved women are diabetic (27%) while 22% of the participants are men with diabetes.

**Figure 2:**
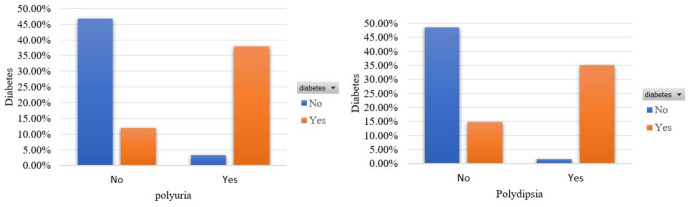
Participants’ distribution in terms of polyuria and polydipsia in the balanced dataset.

In Figure 2, it is shown the participants’ distribution in terms of the features that capture the signs of polyuria and polydipsia. A total of 38% and 35% of participants who suffer from diabetes occur these symptoms. Furthermore, a small percentage of 3.28% and 1.6%, respectively, mentioned these signs although they were not diabetics.

**Figure 3:**
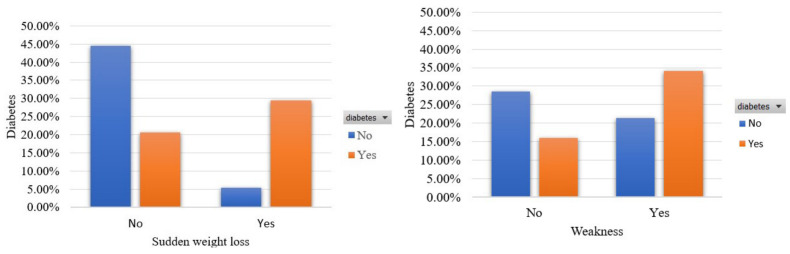
Participants’ distribution in terms of sudden weight loss and weakness in the balanced dataset.

In Figure 3, we demonstrate the participants’ distribution in terms of the features that represent sudden weight loss and weakness. A total of 29% and 34% of participants were diagnosed with diabetes and noted the manifestation of these symptoms, respectively. Furthermore, a percentage of 5.47% and a higher portion of 21.41%, respectively, referred to these signs although they were not diabetics.

**Figure 4:**
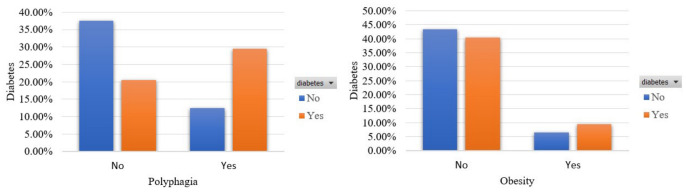
Participants’ distribution in terms of polyphagia and obesity in the balanced dataset.

Figure 4 illustrates the participants’ distribution in terms of the features that denote polyphagia and obesity. A total of 29.53% and 9.53% of participants are diabetics and declared an increase in appetite and that they are obese. In addition, a moderate percentage of 12.50% and a small portion of 6.56% mentioned excessive hunger and obesity, respectively, although they are not diabetics.

**Figure 5:**
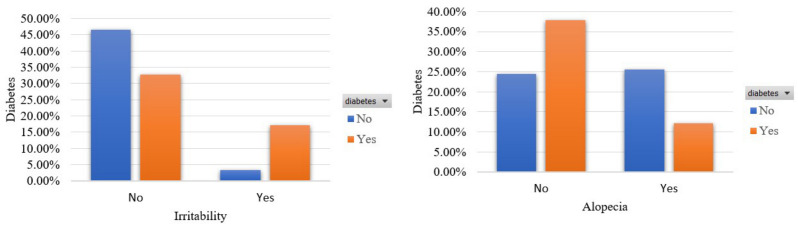
Participants’ distribution in terms of irritability and alopecia in the balanced dataset.

In the following, Figure 5 depicts the irritability and alopecia signs in terms of the involved classes. We see that irritability and alopecia coexist with diabetes in 17.19% and 12.19% of the participants, correspondingly. However, an important portion of 25.63% noted the occurrence of alopecia although they were not diabetic.

**Figure 6:**
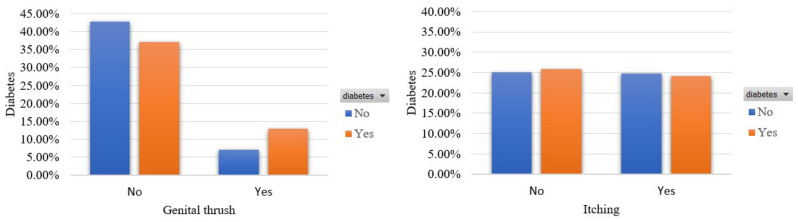
Participants’ distribution in terms of genital thrush and itching in the balanced dataset.

Moreover, Figure 6 presents the occurrence of genital thrush and itching signs in terms of the two classes. We see that these features coexist with diabetes in 12.97% and 24.06% of the participants, correspondingly. However, an important portion of 24.84% noted the occurrence of itching while 7.19% had genital thrush although they were not diabetic.

**Figure 7:**
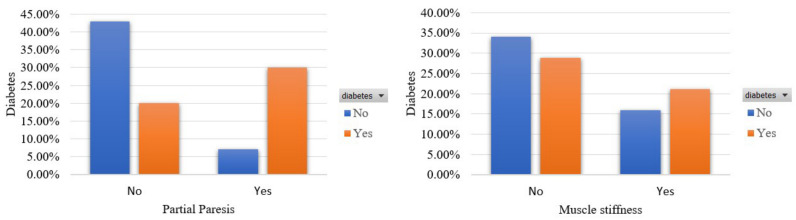
Participants’ distribution in terms of partial paresis and muscle stiffness in the balanced dataset.

Furthermore, Figure 7 focuses on two other diabetes-related symptoms and specifically partial paresis and muscle stiffness. It is observed that 30% and 21% of the involvers are diabetic and manifested theses signs, respectively.

**Figure 8:**
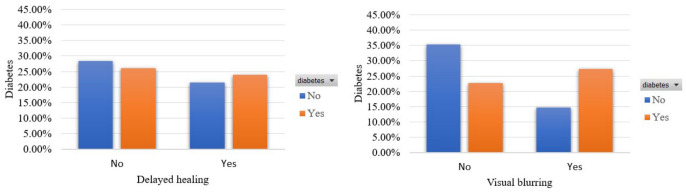
Participants’ distribution in terms of delayed healing and visual blurring in the balanced dataset.

Finally, Figure 8 shows the prevalence of diabetes in terms of the features that capture the occurrence of delayed healing and visual blurring. A total of 50% of those who have been diagnosed with diabetes (or 25% of the total participants) occur visual blurring, which owes to the quick change of blood sugar levels from normal to high. Similar outcomes hold for the coexistence of diabetes and the sign that concern the delay in wound healing, which relate to problems with the immune system activation.

### 3.3. Machine-Learning Models

This subsection will provide a brief description of the ML classification models we relied on for the topic under consideration. Specifically, Naive Bayes, Bayesian Network, Support Vector Machine, Logistic Regression, Artificial Neural Network, K-Nearest Neighbors, J48, Logistic Model Tree, Random Forest, Random Tree, Reduced Error Pruning Tree, Rotation Forest, AdaBoostM1 and Stochastic Gradient Descent were selected in order to evaluate their prediction performance. Here, we note that we assume that each instance *i* in the dataset is represented by a features vector $x_{i}={\left[\begin{array}{c} x_{i1},x_{i2},x_{i3},\cdots ,x_{in} \end{array}\right]}^{T}$, where *n* is the number of the features.

#### 3.3.1. Naive Bayes

Naive Bayes (NB) [57] classifies an instance $x_{i}$ at that class *c* for which $P\left(c\right|x_{i1},\ldots ,x_{in})$ is maximized (under the assumption that the features are highly independent). The conditional probability is defined as $P\left(c\right|x_{i1},\ldots ,x_{in})=\frac{P\left(x_{i1},\ldots ,x_{in}|c\right)P\left(c\right)}{P\left(x_{i1},\ldots ,x_{in}\right)}$, where $P\left(x_{i1},\ldots ,x_{in}|c\right)=\prod_{j=1}^{n}P\left(x_{ij}|c\right)$ is the features probability given class and $P\left(x_{i1},\ldots ,x_{in}\right),P\left(c\right)$ are the prior probability of features and class, respectively. The estimated class is derived by maximizing $P\left(c\right)\prod_{j=1}^{n}P\left(x_{ij}|c\right)$, where c∈{Diabetes,Non−Diabetes}.

#### 3.3.2. Bayesian Network

Bayesian networks (BayesNet) [58] are a widely-used class of probabilistic graphical models. They consist of two parts: a structure and parameters. The structure is a directed acyclic graph (DAG) over a set of features *U* that expresses conditional independencies and dependencies among random variables associated with nodes. The parameters consist of conditional probability distributions associated with each node. A Bayesian network classifier calculates $\arg\max_{c}P(c|x)$ using pa(x) (the set of parents of x∈U) and the distribution P(U) represented by the Bayesian network, based on

(1) $$P\left(c|x\right)=P\left(U\right)/P\left(x\right)\propto P\left(U\right)=\prod_{x\in U}p\left(x|pa\left(x\right)\right).$$

#### 3.3.3. Support Vector Machine

Support Vector Machine (SVM) [59] is used for classification as well as Regression problems. However, primarily, it is used for classification problems in ML. The goal of the SVM algorithm is to create the best line or decision boundary that can segregate n-dimensional space into classes so that we can easily put the new data point in the correct category in the future. This best decision boundary is called a hyperplane. Support Vector Machine (SVM) finds the hyperplane that can optimally separate instances into two classes. The most characteristic Kernel functions are linear, polynomial, radial basis and quadratic. An instance $x^{\prime }$ can be optimally classified based on function:

(2) $$\begin{array}{c} f\left(x^{\prime }\right)=Sgn\left[\sum_{i=1}^{M}\alpha_{i}c_{i}K\left(x_{i},x^{\prime }\right)+b\right]\\ 0\leq \alpha_{i}\leq C,\;\sum \alpha_{i}c_{i}=0,\;\alpha_{i}\geq 0,i=1,2,\cdots ,M \end{array}$$

where *M* is the size of training instances, $x_{i},c_{i}$ are the training instance feature vector and its class label, respectively, *b* is a bias, $c_{i}\in \left\{1,\;-1\right\}$, $K(x_{i},x^{\prime })$ is the kernel function which corresponds the input vectors into an expanded feature space.

#### 3.3.4. Logistic Regression

Logistic regression (LR) [60] is one of the most popular ML algorithms, which comes under the Supervised Learning technique. It is used for predicting the categorical dependent variable using a given set of independent features. Logistic regression predicts the class output, which can be either Yes or No (0 or 1). The probability an instance to belong in the Diabetes class is *p*, thus, 1−p is the probability of an instance belonging to the Non-Diabetes class. The relationship of log-odds with base *b* and model parameters $\beta_{i}$ is written as:

(3) $$log_{b}\left(\frac{p}{1-p}\right)=\beta_{0}+\beta_{1}x_{i1}+\cdots +\beta_{n}x_{in}$$

#### 3.3.5. Artificial Neural Network

A fully connected multi-layer neural network is called a Multilayer Perceptron (MLP) [61]. It consists of three types of layers, such as the input layer, output layer and hidden layer. The MLPs are designed to approximate any continuous function and can solve problems that are not linearly separable. Furthermore, it can use any arbitrary activation function.

#### 3.3.6. K-Nearest Neighbors

The K-nearest neighbors algorithm (KNN) [62] is a non-parametric, supervised learning classifier that uses proximity to make classifications or predictions about the grouping of an individual data point.

#### 3.3.7. J48

J48 [63] is a machine-learning decision tree classification algorithm that examines the data categorically and continuously. It deals with the problems of the numeric attributes, missing values, pruning, estimating error rates, the complexity of decision tree induction and generating rules from trees.

#### 3.3.8. Logistic Model Tree

A logistic model tree (LMT) [64] consists of a standard decision tree structure with logistic regression functions $f\left(x_{i}\right)=\beta_{0}+\sum_{j=1}^{n}\left(\beta_{i}x_{ij}\right)$ at the leaves. LMT produces a single tree containing binary splits on numeric attributes, multiway splits on nominal ones and logistic regression models at the leaves, and the algorithm ensures that only relevant attributes are included in the latter.

#### 3.3.9. Random Forest

Random Forest (RF) [65] is a popular ML algorithm that belongs to the supervised learning technique. It is used in classification and regression problems. It builds decision trees on different samples and takes their majority vote for classification and average in case of regression.

#### 3.3.10. Reduced Error Pruning Tree

Reduced Error Pruning Tree (RepTree) [66] is a fast decision tree learner that builds a decision/regression tree using information gain as the splitting criterion and prunes it using a reduced error pruning algorithm.

#### 3.3.11. Random Trees

Random Tree (RT) [67] is an ensemble of multiple decision trees. The Random Trees node is built on the Classification and Regression Tree methodology. It splits the training records (through recursive partitioning) into segments with similar output features’ values. The node initially examines the available input features in order to find the best split evaluating the impurity index. All splits are binary.

#### 3.3.12. Rotation Forest

Rotation Forest (RotF) [68] is a method for generating classifier ensembles based on feature extraction. In order to create the training data for a base classifier, the feature set is randomly split into subsets, and principal component analysis (PCA) is applied to each subset.

#### 3.3.13. AdaBoostM1

Let $G_{m}\left(x_{i}\right)$, for m=1,2,⋯,M, be the sequence of weak classifiers. Our objective is to build the $G\left(x\right)=sign(\sum_{m=1}^{M}\alpha_{m}G_{m}\left(x_{i}\right))$. The final prediction is a combination of the predictions from all classifiers through a weighted majority vote. At the first step, m=1, the weights are initialized uniformly $w_{l}=1/N$. The coefficients $\alpha_{m}$ are computed by the boosting algorithm and weight the contribution of each respective $G_{m}\left(x_{i}\right)$ giving higher influence to the more accurate classifiers in the sequence. At each boosting step, the data is modified by applying weights $w_{1},w_{2},\cdots ,w_{N}$ to each training observation. At step *m*, the observations that were misclassified previously have their weights increased [69].

#### 3.3.14. Stochastic Gradient Descent

Stochastic gradient descent (SGD) [70] is an efficient approach to fitting linear classifiers and regressors under convex loss functions, such as linear SVM and LR. The SGD has been successfully applied to large-scale and sparse machine learning problems.

#### 3.3.15. Stacking

Stacking is a common approach that is utilized to acquire more accurate predictions than single models’. Stacking uses the predicted class labels of the base models as input features to train a meta-classifier that undertakes to find the class label [71].

### 3.4. Evaluation Metrics

In this research work, various metrics, such as the accuracy, precision, recall, F-Measure and AUC [72], are examined in order to evaluate the performance of the machine-learning models. Each metric will help us to identify the strengths and weaknesses of the models. The desired metrics are calculated with the help of the Confusion matrix. The confusion matrix consists of the elements true positive (TP), true negative (TN), false positive (FP) and false-negative (FN). Performance metrics are defined as

(4) $$\begin{array}{ccccc} Precision=\frac{TP}{TP+FP}, & \; & Recall=\frac{TP}{TP+FN} & \; & \end{array}$$

(5) $$\begin{array}{ccc} F-Measure=2\frac{Precision\cdot Recall}{Precision+Recall}, & \; & Accuracy=\frac{TN+TP}{TN+TP+FN+FP} \end{array}$$

Precision indicates how many of those who are labeled as diabetic actually belong to this class. Recall shows how many of those who are diabetic are correctly predicted. F-Measure is the harmonic mean of the precision and recall and captures the predictive performance of a model. The Accuracy illustrates the proportion of the total number of predictions that were correct.

To evaluate the distinguishability of a model, the Area under curve (AUC) is exploited. It is a metric that varies in [0, 1]. The closer to one, the better the ML model performance is in distinguishing diabetes from non-diabetes instances. If AUC equals one, the ML model can perfectly separate the instances distribution of two classes. In special case where all non-diabetes (diabetes) are classified as diabetes (non-diabetes), the AUC equals 0.

## 4. Results and Discussion

### 4.1. Experiments Setup

The machine-learning models’ performance is evaluated in the Waikato Environment for Knowledge Analysis (Weka) [73]. It is developed at the University of Waikato, New Zealand and is free software. Furthermore, it provides a library of various models for data preprocessing, classification, clustering, forecasting, visualization, etc. The computing system in which the experiments were conducted has the following characteristics: 11th Gen Intel(R) Core(TM) i7-1165G7 @ 2.80 GHz 2.70 GHz, 16 GB, Windows 11 Home, 64-bit Operating System and x64-based processor. For our experiments, 10-fold cross-validation and percentage split (80:20) were applied to measure the models’ efficiency in the balanced dataset of 640 instances. In Table 3, the parameters’ settings of the considered models are shown.

### 4.2. Evaluation

In this research work, various ML models, such as BayesNet, NB, SVM, LR, ANN, KNN, J48, LMT, RF, RT, RepTree, RotF, AdaBoostM1 and SGD and Ensemble method (Stacking), are evaluated in terms of the accuracy, precision, recall, F-measure and AUC.

In Table 4, we illustrate the performance of the models under consideration after applying SMOTE with 10-fold cross-validation. From the results of the experiments, we can see that the KNN and RF models present the best prediction accuracy with 98.59% compared to the corresponding proposed models. Furthermore, the RotF and RF models have an AUC of 99.9%. It should be noted that in SMOTE with 10-fold cross-validation, all our models have an accuracy greater than 88.75% (BayesNet) and an AUC greater than 94.2% (SGD).

Moreover, in Table 5, we summarize related works based on the dataset [36] after applying 10-fold cross-validation on the same features we relied on but without SMOTE. Our proposed models after SMOTE and 10-fold cross-validation showed better performance in terms of accuracy compared to the related works as shown in Table 5.

In addition, in Table 6, we depict the performance of ML models in terms of accuracy, recall, precision, F-measure and AUC after applying SMOTE and percentage split (80:20). Both in this case, the KNN and RF achieved the best performance in relation to the rest models with an accuracy of 99.22%. Furthermore, the RF model and the Stacking method performed an AUC of 100%. Our proposed models have excellent AUC rates greater than 93.7% (SGD) and accuracy greater than 88.28% (BayesNet).

Furthermore, in Table 7, we outline the accuracy of our proposed models, such as NB, LR J48 and RF, after applying SMOTE and percentage split (80:20). The same table shows the results of the work [32] after applying a percentage split (80:20) on the same features we relied on but without SMOTE. We observe that our proposed models showed better accuracy but with a small percentage gap of 0.22–1.97%.

Finally, we note a limitation of this research work. This study was based on a publicly available dataset. The dataset we relied on does not come from a hospital unit or institute, which could give us richer information data models with different characteristics, such as biochemical measurements that record a detailed health profile of the participants. Acquiring access to such data is time-consuming and difficult for privacy reasons.

## 5. Conclusions

The habits and lifestyle of the modern world are the results of the growing incidence of diabetes. Medical professionals now have the opportunity, with the contribution of machine-learning techniques, to assess the relative risk and provide appropriate guidelines and interventions for the management and treatment or prevention of diabetes.

In this research article, we applied several machine-learning models in order to identify individuals at risk of diabetes based on specific risk factors. Data exploration through risk factor analysis could help to identify associations between the features and diabetes. Performance analysis showed that data pre-processing is a major step in the design of efficient and accurate models for diabetes occurrence.

Specifically, after applying SMOTE with 10-fold cross-validation, the Random Forest and KNN outperformed the other models with an accuracy of 98.59%. Similarly, applying SMOTE with a percentage split (80:20), the Random Forest and KNN outperformed the other models with an accuracy of 99.22%. In both cases, applying SMOTE, our proposed models were superior to the related published research works based on the [36] dataset with the same features we relied on in terms of accuracy.

In future work, we aim to extend the machine-learning framework through the use of deep-learning methods by applying a Long-Short-Term-Memory (LSTM) algorithm and Convolutional Neural Networks (CNN) in the same dataset and comparing the results in terms of accuracy with relevant published works.

## Figures and Tables

**Table 1:** Related works for the subject under consideration.

Research Work	Use Case	Dataset	Proposed Models	Metrics
[26]	Diabetes Prediction	Pima Indian Diabetes Dataset	Soft Weighted Voting	AUC: 0.950 Sensitivity: 0.789 Specificity: 0.934
[27]	Diabetes Classification	Pima Indian Diabetes Dataset	SVM/KNN	SVM: Accuracy 0.89, Precision 0.88 KNN: Recall 0.9, F1 score 0.88
[28]	Diabetes Detection	Not Publicly Available	Simple Linear Regression	RMSE: 0.838
[29]	Diabetes Prediction	Pima Indian Diabetes Dataset	Random Forest	Accuracy: 94.1%
[30]	Classification and prediction of diabetes	National Health and Nutrition Examination Survey	Random Forest	Accuracy: 94.25% AUC: 0.95
[31]	Diabetes Detection	ELSA Database	Weighted Majority Voting	AUC: 0.884
[32]	Diabetes Prediction	[36]	Random Forest	Accuracy: 94.1% 10-fold cross-validation Accuracy: 99% Percentage split (80:20)
[33]	Diabetes Prediction	[36]	KNN	Accuracy: 98.07%
[34]	Diabetes Prediction	[36]	Random Forest	Accuracy, Precision, Recall, F-Measure: 97.5%
[35]	Diabetes Prediction	[36]	Random Forest	Accuracy: 97.88%

**Table 2:** Evaluation of feature importance based on the Pearson Correlation, Gain Ratio, Naive Bayes and Random Forest.

Feature	Pearson Rank	Feature	Gain Ratio	Feature	Naive Bayes (AUC)	Feature	Random Forest (AUC)
polyuria	0.7046	polydipsia	0.4317	polyuria	0.3329	polyuria	0.3337
polydipsia	0.6969	polyuria	0.4143	polydipsia	0.3189	polydipsia	0.3189
sudden_weight_loss	0.5017	gender	0.2117	sudden_weight_loss	0.2229	age	0.2537
gender	0.4922	sudden_weight_loss	0.2088	gender	0.2089	sudden_weight_loss	0.2232
partial_paresis	0.4757	partial_paresis	0.1814	partial_paresis	0.2084	gender	0.2092
polyphagia	0.3450	irritability	0.1218	polyphagia	0.1454	partial_paresis	0.2084
irritability	0.3398	polyphagia	0.0895	irritability	0.1174	polyphagia	0.1456
alopecia	0.2771	alopecia	0.0588	alopecia	0.1099	irritability	0.1175
visual_blurring	0.2564	age	0.0533	visual_blurring	0.1098	alopecia	0.1118
weakness	0.2547	visual_blurring	0.0489	weakness	0.1093	visual_blurring	0.1103
genital_thrush	0.1441	weakness	0.0477	age	0.0584	weakness	0.1096
age	0.1124	genital_thrush	0.0209	genital_thrush	0.0468	genital_thrush	0.0471
muscle_stiffness	0.1068	muscle_stiffness	0.0086	muscle_stiffness	0.0324	muscle_stiffness	0.0327
obesity	0.0808	obesity	0.0074	obesity	0.0180	obesity	0.0191
delayed_healing	0.0471	delayed_healing	0.0016	delayed_healing	0.0046	delayed_healing	0.0049
itching	0.0156	itching	0.0002	itching	−0.0273	itching	−0.0260

**Table 3:** Machine Learning models’ settings.

Models	Parameters
**BayesNet**	estimator: simpleEstimator searchAlgorithm: K2 useADTree: False
**NB**	useKernelEstimator: False useSupervisedDiscretization: False
**SVM**	eps = 0.001 gamma = 0.0 kernel type: radial basis function loss = 0.1
**LR**	ridge = $10^{-8}$ useConjugateGradientDescent: False
**ANN**	hidden layers: ‘a’ learning rate = 0.3 momentum = 0.2 training time = 500
**KNN**	K = 1 Serach Algorithm: LinearNNSearch with Euclidean
**J48**	reducedErrorPruning: False savelnstanceData: False subtreeRaising: True
**LMT**	errorOnProbabilities: False fastRegression: True numInstances = 15 useAIC: False
**RF**	maxDepth = 0 numIterations = 100 numFeatures = 0
**RT**	maxDepth = 0 minNum = 1.0 minVarianceProp = 0.001
**RepTree**	maxDepth = −1 minNum = 2.0 minVarianceProp = 0.001
**RotF**	classifier: J48 numberOfGroups: False projectionFilter: PrincipalComponents
**AdaBoostM1**	classifier: DecisionStump resume: False useResampling: False
**SGD**	epochs = 500 epsilon = 0.001 lamda = $10^{-4}$ learningRate = 0.01 lossFunction: Hinge loss (SVM)
**Stacking**	Base Models: RF, KNN Meta-model:LR

**Table 4:** Performance evaluation after SMOTE with 10-fold cross-validation.

	Accuracy	Precision	Recall	F-Measure	AUC
**BayesNet**	88.75 ± 5.04%	88.9 ± 4.8%	88.8 ± 4.9%	88.7 ± 5.1%	95.6 ± 2.1%
**NB**	88.91 ± 5.02%	89.1 ± 4.7%	88.9 ± 5%	88.9 ± 5.1%	95.5 ± 2.4%
**SVM**	95.62 ± 2.06%	95.7 ± 1.8%	95.6 2.1%	95.6 ± 2.1%	95.6 ± 2.1%
**LR**	93.44 ± 2.64%	93.4 ± 2.6%	93.4 ± 2.6%	93.4 ± 2.7%	97.6 ± 1.4%
**ANN**	96.45 ± 2.00%	97.3 ± 2.40%	97.3 ± 2.40%	97.2 ± 2.30%	99.1 ± 2.60%
**KNN**	98.59 ± 1.72%	98.6 ± 1.62%	98.6 ± 1.70%	98.6 ± 1.70%	98.9 ± 1.30%
**J48**	97.19 ± 2.74%	97.2 ± 2.70%	97.2 ± 2.70%	97.2 ± 2.70%	97.2 ± 2.20%
**LMT**	97.19 ± 1.61%	97.2 ± 1.60%	97.2 ± 1.60%	97.2 ± 1.60%	98.3 ± 1.30%
**RF**	98.59 ± 1.15%	98.6 ± 1.10%	98.6 ± 1.12%	98.6 ± 1.12%	99.9 ± 0.20%
**RT**	97.97 ± 2.09%	98 ± 2.10%	98 ± 2.10%	98 ± 2.10%	98 ± 2.10%
**RepTree**	93.12 ± 3.23%	93.2 ± 3.00%	93.1 ± 3.20%	93.1 ± 3.20%	96.4 ± 2.30%
**RotF**	98.28 ± 2.01%	98.3 ± 1.17%	98.3 ± 2.00%	98.3 ± 2.00%	99.9 ± 0.20%
**AdaBoostM1**	90.78 ± 2.59%	91.2 ± 2.40%	90.8 ± 2.60%	90.8 ± 2.60%	97.1 ± 2.10%
**SGD**	94.22 ± 2.56%	94.3 ± 2.40%	94.2 ± 2.60%	94.2 ± 2.60%	94.2 ± 2.60%
**Stacking**	98.49 ± 1.10%	98.5 ± 1.10%	98.5 ± 1.11%	98.5 ± 1.11%	99.7 ± 0.20%

**Table 5:** Model comparison in terms of accuracy with 10-fold cross-validation.

	Accuracy				
	Proposed models	[32]	[33]	[34]	[35]
**BayesNet**	88.75%	-	86.92%	-	-
**NB**	88.91%	87.4%	87.11%	87.1%	-
**SVM**	95.62%	-	92.11%	92.1%	-
**LR**	93.44%	92.4%	-	-	-
**ANN**	96.45%	-	-	96.3%	96.34%
**KNN**	98.59%	-	98.07%	-	-
**J48**	97.19%	95.6%	95.96%	-	-
**RF**	98.59%	97.4%	97.5%	97.5%	97.88%
**RT**	97.97%	-	96.15%	-	-

**Table 6:** Performance evaluation after SMOTE with percentage split (80:20).

	Accuracy	Precision	Recall	F-Measure	AUC
**BayesNet**	88.28%	88.3%	88.3%	88.3%	95.9%
**NB**	89.06%	89.1%	89.1%	89.1%	95.8%
**SVM**	97.66%	97.7%	97.7%	97.7%	97.6%
**LR**	92.97%	93%	93%	93%	98.5%
**ANN**	97.66%	97.7%	97.7%	97.7%	99.9%
**KNN**	99.22%	99.2%	99.2%	99.2%	98.9%
**J48**	95.53%	95.5%	95.5%	95.5%	96.1%
**LMT**	96.87%	96.9%	96.9%	96.9%	99.4%
**RF**	99.22%	99.2%	99.2%	99.2%	100%
**RT**	97.66%	97.7%	97.7%	97.7%	97.7%
**RepTree**	92.19%	92.2%	92.2%	92.2%	95.2%
**RotF**	97.66%	97.7%	97.7%	97.7%	99.9%
**AdaBoostM1**	92.97%	93%	93%	93%	97.5%
**SGD**	93.75%	93.8%	93.8%	93.8%	93.7%
**Stacking**	99.20%	99.2%	99.2%	99.2%	100%

**Table 7:** Model comparison in terms of accuracy with percentage split (80:20).

Accuracy				
	**NB**	**LR**	**J48**	**RF**
Proposed models	89.06%	92.97%	95.53%	99.22%
[32]	88%	91%	95%	99%
